# Upregulation of astrocytic α7 nicotinic receptors in Alzheimer's disease brain- possible relevant to amyloid pathology

**DOI:** 10.1186/1750-1326-7-S1-O7

**Published:** 2012-02-07

**Authors:** Wenfeng Yu, Naguib Mechawar, Slavica Krantic, Jean-Guy Chabot, Rémi Quirion

**Affiliations:** 1 Department of Psychiatry, Douglas Mental Health University Institute, McGill University, Montréal, Quebec, Canada

## Background

Alzheimer’s disease (AD) is characterized by extracellular deposits of β-amyloid (Aβ), intracellular neurofibrillary tangles and inflammation response. The α7 nicotinic receptors (nAChRs) were proposed to be directly relevant to the pathogenesis of AD by interacting with Aβ. The α7 nAChRs and Aβ1-42 are co-localized in neuritic plaques and neurons and can form a high affinity stable complex. The up-regulation of α7 nAChRs on activated astrocytes has been reported in the AD brain. However, because of the poor specificity of currently available α7 nAChR antibodies, it is important to develop new ways to investigate the discrete distribution of α7 nAChRs in nervous tissue.

## Material and methods

α7 nAChR single immunocytochemistry, double and triple-labeling immunocytochemistry were performed on formalin-fixed paraffin-embedded sections from 8 brain samples of neuropathologically confirmed sporadic AD and age-matched controls. Tissues were obtained from the Quebec Brain Bank (QBB; Douglas Institute, Montréal, QC, Canada). Immunohistochemical staining was performed as described previously (Yu et al., 2005). Primary cortical and hippocampal astrocytic cultures were prepared from the brains of newborn mouse (129S6 strain) according to the procedure of McCarthy and de Vellis (1980). The purified astrocytes were exposed to 100 nM Aβ_1–42_ oligomers for 24h at 37 C with or without co-treatment with a series of concentration of PNU282987 and S24795 for 12 h. To inhibit α7 nAChR activities, cells were pretreated with methyllycaconitine (MLA) for 45 min before Aβ_1–42_ oligomers/PNU282987/S24795. After Aβ_1–42_ treatments for 24 hours, the culture media were collected and was concentrated using Centricon YM-3 (Millipore, Temecula, CA). To prepare cell lysates, the treated cells were rinsed with 1× PBS, quick frozen then lysed on ice for 30 min in a lysis buffer. The lysates were then centrifuged at 10,000 × *g* for 15 min, and the supernatant was collected. Protein concentrations were determined and equalized before samples were applied to western blotting for measuring Aβ oligomers.

## Results

We successfully characterized the distribution of α7 nAChRs in AD brains using biotin conjugated α-bungarotoxin. Our morphological data show the upregulation of α7 nAChRs in activated astrocytes in the AD brain. A certain proportion of α7 nAChRs positive astrocyes were morphologically associated with amyloid plaques. In mouse primary cultured astrocytes, treatments with different α7 nAChR agonists (PNU282987, S24795) significantly enhanced astrocytic Aβ phagocytosis and inhibited Aβ1-42 aggregation.

## Conclusion

Take together, our study suggest that astrocytic α7 nAChRs possibly contribute to the initiation and development of amyloid pathology in the AD brain and thus should be considered as a potential therapeutic target, in addition to the better characterized neuronal nicotinic receptors.

